# Streamlining eligibility assessment for Alzheimer's disease-modifying therapies: Prediction of MMSE scores using the digital clock and recall

**DOI:** 10.3389/fdgth.2026.1799372

**Published:** 2026-07-09

**Authors:** Ali Jannati, Claudio Toro-Serey, Marissa Ciesla, Emma Chen, John Showalter, David Bates, Alvaro Pascual-Leone, Sean Tobyne

**Affiliations:** 1 Department of Neurology, Harvard Medical School, Boston, MA, United States; 2 Linus Health, Inc., Boston, MA, United States; 3 Hinda and Arthur Marcus Institute for Aging Research and Deanna and Sidney Wolk Center for Memory Health, Hebrew SeniorLife, Boston, MA, United States

**Keywords:** Alzheimer's disease, dementia, digital clock and recall, digital cognitive assessment, disease-modifying treatment, machine learning, mild cognitive impairment, mini-mental state examination

## Abstract

**Introduction:**

The eligibility of anti-amyloid disease-modifying therapies (DMTs) and their integration into clinical practice in some institutions requires a specific range of Mini-Mental State Examination (MMSE) scores. Reliance on this pencil-and-paper psychometric instrument imposes operational burdens and risks of perpetuating health disparities, given the test's known educational and cultural biases. This study evaluates the efficacy of the Digital Clock and Recall (DCR™)—a rapid, FDA-listed digital cognitive assessment—to crosswalk to MMSE scores using machine learning, thereby offering a faster, scalable, and equitable mechanism for patient triage.

**Methods:**

We conducted a retrospective analysis using data from the multi-site Bio-Hermes-001 (BH) study (*N*CT04733989, *N* = 945). Participants were clinically classified as cognitively unimpaired, mild cognitive impairment, or probable Alzheimer's dementia. We trained a Poisson elastic net regression model on 70% of the sample, using age and multimodal digital features derived from the DCR (including drawing kinematics and voice acoustics) to predict MMSE scores. The model was validated using the remaining 30% of Bio-Hermes-001 and an independent external validation cohort from the Apheleia study (NCT05364307, *N* = 238).

**Results:**

The machine learning model predicted MMSE scores with a root-mean-squared error (RMSE) of 2.43 in the BH test set. This error margin falls within the established test-retest reliability range of the manual MMSE itself (∼4.0–4.2 points at short inter-test intervals), providing evidence that the predicted score is of comparable precision to a repeat human administration of the MMSE. External validation in the Apheleia cohort demonstrated robust generalizability (RMSE = 2.62). In the BH held-out test set, the model showed comparable performance across Race (White RMSE = 2.46; Non-White RMSE = 2.25) and Ethnicity (Hispanic RMSE = 2.19; Non-Hispanic RMSE = 2.45), a balanced pattern also observed in the Apheleia-001 external cohort. Exploratory demographic analyses on prediction errors, including Age, Sex, Race, and Ethnicity, yielded significant differences only for Sex and Age in Apheleia, with signed errors becoming progressively more negative (i.e., increasing under-prediction) at older ages for the latter. This scarcity of statistical differences across cohorts suggested that our predictions were fair.

**Discussion:**

Machine learning can leverage multimodal features from the DCR to accurately and equitably crosswalk to MMSE scores in support of current guidelines, transforming a time-intensive manual test into a rapid, automated assessment. By deploying this “digital triage” engine, where traditional assessments are still used for DMT eligibility, healthcare systems can streamline the identification of DMT-eligible patients, reduce specialist referral bottlenecks, and ensure that access to life-altering therapies is determined by pathology rather than demography.

## Introduction

The recent regulatory approval of anti-amyloid disease-modifying therapies (DMTs), specifically lecanemab (Leqembi®) and donanemab (Kisunla®), marks a historic inflection point in the management of patients with Alzheimer's disease (AD) (1, 2). For the first time, clinicians possess pharmacological treatments capable of slowing the clinical progression of mild cognitive impairment (MCI) or early dementia due to AD by targeting underlying amyloid pathology. However, the determination of eligibility for DMTs and their successful integration into clinical practice at some institutions is currently impeded by the reliance on legacy cognitive-screening tests, most notably the Mini-Mental State Examination (MMSE). The pivotal clinical trials that ultimately supported the Food and Drug Administration (FDA) approval of lecanemab and donanemab, i.e., CLARITY AD and TRAILBLAZER-ALZ 2, utilized specific MMSE score ranges (23–29 and 20–28, respectively) as key inclusion criteria, positioning these scores as indicators of eligibility post-market (3, 4). Consequently, millions of older adults must be screened to identify the subset eligible for confirmatory biomarker testing (e.g., amyloid PET or CSF analysis) and subsequent therapy. The current primary care infrastructure is ill-equipped to handle this volume. The MMSE, while commonly used, requires 10–15 min of direct clinician administration—a prohibitive cost in primary care settings where visit times average less than 15 min (5). Furthermore, the test is subject to significant inter-rater variability and “ceiling effects” that can mask early impairment in high-functioning individuals (6, 7).

More critically, the MMSE is structurally biased against individuals from underrepresented racial and ethnic groups and those with lower educational attainment. Studies have consistently demonstrated that Black and Hispanic older adults are more likely to be misclassified as impaired by the MMSE compared to White counterparts with similar levels of pathology, largely due to cultural and educational nuances in test items (8, 9). Relying on such a biased instrument for gatekeeping high-value care threatens to exacerbate systemic healthcare inequities, potentially denying life-altering treatment to disadvantaged populations.

To address these challenges, the field is increasingly turning to digital cognitive Assessments (DCAs). Linus Health's Digital Clock and Recall (DCR™) represents a technological evolution of the Mini-Cog (10), using a digitizing, commercially available tablet to capture the process of completing a cognitive task with millisecond precision, rather than considering only the final product. By analyzing nearly 2,000 process-based kinematic, temporal, acoustic, and speech features, such as processing speed, hesitation, and motor planning, the DCR offers a more sensitive, objective, and less biased measure of cognitive function than traditional pen-and-paper scores (10–15). However, the clinical utility of such DCAs depends on their ability to interface with existing regulatory frameworks and the way clinicians adopt them in practice. Despite an ever-growing body of literature detailing the superiority of DCAs over traditional assessments, clinicians may be reluctant to replace long-standing, familiar instruments quickly. Providing a validated score “crosswalk” between a new DCA and legacy measures [e.g., MMSE or Montreal Cognitive Assessment (MoCA)] can improve interpretability and support incremental adoption while clinical workflows evolve to leverage newer, superior tools (10, 11, 16–18).

Although a comprehensive review of prior digital cognitive assessment research is beyond the scope of a Brief Research Report, several bodies of work directly inform the present study. First, prior statistical crosswalks between legacy scales have been constructed for the MoCA and MMSE (17), providing a precedent for inter-instrument translation that the present work extends to a digital biomarker. Second, digital clock drawing metrics and DCR-derived features have been associated with amyloid and tau burdens (13, 19–22), thereby establishing the biological validity of the features used here. Third, the DCR has been evaluated in a range of real-world implementations and comparative settings, including comparisons with the MoCA and SLUMS in primary care (23, 24), deployment in acute ischemic stroke care (25), and global implementation within the Davos Alzheimer's Collaborative early detection program (26, 27). Fourth, to our knowledge, this is the first externally validated machine-learning crosswalk from multimodal process-based DCR features to the MMSE with an explicit evaluation of demographic fairness across both the training and independent validation cohorts. Beyond DMT eligibility, such a crosswalk supports backward compatibility of digital biomarkers with the large archive of legacy MMSE-based datasets, enabling longitudinal and cross-cohort comparisons as the field transitions away from pencil-and-paper administration.

This study presents the development and validation of a machine learning (ML) model that predicts MMSE scores using multimodal features derived from the DCR. Leveraging data from a large multi-site study and an external validation cohort, we demonstrate that an ML model based on DCR features can predict MMSE scores with accuracy comparable to the test-retest reliability of the MMSE itself. By providing a rapid, automated, scalable, and equitable crosswalk for the MMSE, this innovation is designed to streamline “digital triage” for clinicians who currently rely on MMSE scores, improving referral decisions, optimizing access to specialist care, and supporting broader, fairer access in the new era of Alzheimer's therapeutics.

## Methods

Data were obtained from 945 participants enrolled in the multi-site Bio-Hermes-001 study (BH; ClinicalTrials.gov ID: NCT04733989). The study was coordinated by the Global Alzheimer's Platform Foundation (GAP), with protocols implemented at community-based clinical trial sites. This was an observational study designed to build a biomarker database by collecting blood-based biomarker measures and comparing them to brain amyloid status determined by amyloid PET or CSF. Participants also completed a battery of cognitive assessments, including a mix of traditional assessments and DCAs. Participants or their legally authorized representatives provided written informed consent prior to participation.

**Figure 1:**
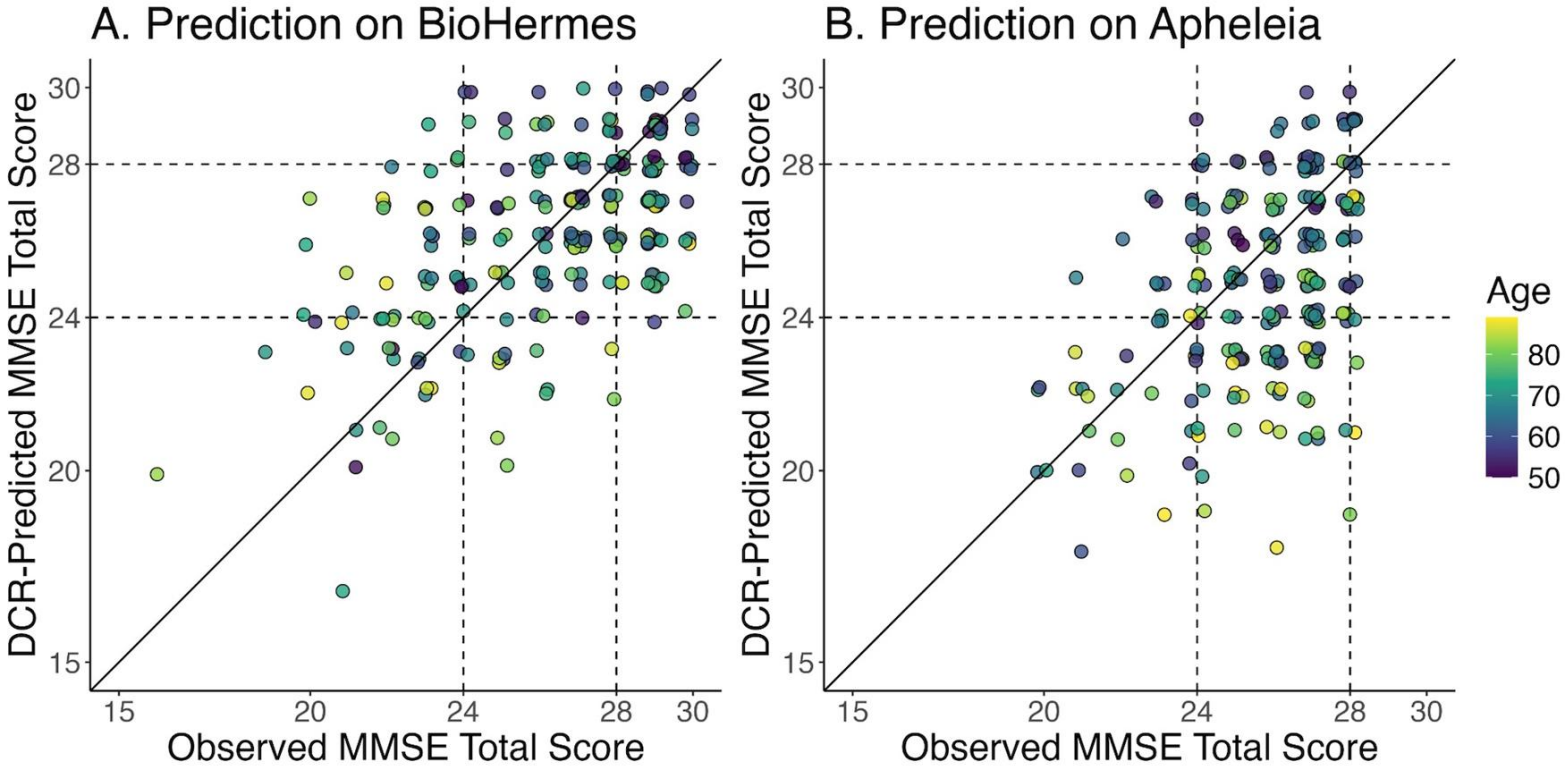
**(A)** predicted vs. actual MMSE total score for an elastic net model of DCR features from the BH data set. Lines indicate MMSE score thresholds denoting putative mild and more severe cognitive impairment. Points are shown with alpha-blending and jitter (height and width = 0.2) to make overlapping observations visible. **(B)** Predicted vs. actual MMSE total score for an elastic net model of DCR features from the Apheleia cohort. Lines indicate MMSE score thresholds denoting putative mild and more severe cognitive impairment. No MMSE scores of 29–30 are observed in this panel because the Apheleia cohort was enrolled based on progressive cognitive concerns and does not include a representative cognitively-unimpaired population (see Methods and Limitations). Predictions in panel **(B)** are rendered at the same continuous (Poisson-exponentiated) resolution as panel **(A)**.

For external validation, we used data from 238 participants in the multisite Apheleia-001 study (ClinicalTrials.gov ID: NCT05364307), coordinated by the Global Alzheimer's Platform Foundation (GAP) in collaboration with AbbVie, Inc. The study evaluated screening strategies, particularly blood-based biomarker testing, intended to reduce screen failures in Alzheimer's disease therapeutic clinical trials. The objective was to identify and characterize individuals reporting memory concerns and/or cognitive impairment using demographics, clinical history, brief cognitive measures, and blood-based biomarkers, thereby increasing the likelihood of trial randomization (28). Written informed consent was obtained from each participant or their legally authorized representative prior to enrollment. Because Apheleia-001 enrolled participants with “progressive cognitive concerns reported by the participant or a caregiver” and excluded those with a prior negative amyloid PET scan, this cohort is by design enriched for memory complaints and does not contain a representative cognitively unimpaired population; this is reflected in the absence of observed MMSE scores of 29–30 in the cohort (see Figure 1B and Limitations).

### Participants and study cohort

#### Bio-Hermes-001 study

Full enrollment criteria have been reported previously (13, 29). The sample had a mean (±SD) age of 72.0 (±6.7) years; 57% were female; 10% identified as Hispanic or Latino; 85% as White, and 11% as Black or African American. Participants had a mean (±SD) of 15.0 (±2.7) years of education, and English was the primary language for all participants (Table 1). Participants were prospectively categorized by the study team as cognitively unimpaired (CU; *n* = 402), mild cognitive impairment (MCI; *n* = 298), or probable Alzheimer's dementia (pAD; *n* = 242). Group assignments were determined at the screening visit (Visit 1) based on MMSE performance (30), the Rey Auditory Verbal Learning Test (RAVLT) (31), and the Functional Activities Questionnaire (FAQ) (32), together with a clinical interview and medical record review conducted by study site personnel. Alternatively, participants were assigned to the MCI or pAD cohorts based on a clinical diagnosis made within three months of Visit 1. Detailed classification criteria are provided in prior work (see Supplementary Materials in (13)). Analyses in the present study used only Visit 1 data, including the DCR. Performance on the DCR was not considered in cohort assignment, and the authors remained blinded to cohort membership until the database lock.

**Table 1:** Demographics for Bio-Hermes-001 and Apheleia-001 studies.

Measure/Study	Bio-Hermes-001				Apheleia-001
	Healthy	MCI	Probable AD dementia	No Diagnosis	–
N	402	298	242	3	238
Sex (% Female)	62	54	53	67	65
Age (Median [SD])	70 (6.48)	72 (6.77)	75 (6.25)	76 (4.73)	68 (10.35)
Years of Education (Median [SD])	16 (2.45)	16 (2.76)	15 (2.99)	18 (2.00)	15 (2.65)
Ethnicity (% Hispanic)	7	11	14	0	19
Race (% White)	87	86	84	100	76
DCR (Median [IQR])	3 (3)	2 (2)	0 (2)	2 (2)	2 (2)

The BH study sample was used to train the model, and the Apheleia sample was used to externally validate it. No cognitive cohorts were available for the Apheleia study.

#### Apheleia-001 study

Eligible participants were 50 to 90 years old (inclusive) and had progressive cognitive concerns reported by the participant or a caregiver. Individuals with a known or self-reported negative amyloid PET scan within the prior 24 months were excluded. Participants were also excluded if they had experienced a stroke within six months of prescreening or had another neurological disorder (aside from AD) that investigators judged could be contributing to cognitive impairment. The sample had a mean (±SD) age of 68.0 (±10.4) years; 65% were female; and 19% identified as Hispanic or Latino. Racial composition was 76% White, 15% Black or African American, 4% Asian, and 4% Other. Participants had a mean (±SD) of 15.0 (±2.7) years of education, and English was the primary language for all participants. All participants completed both the DCR and the MMSE. Beyond administration of the cognitive measures, the study protocol did not assign an overall cognitive diagnostic classification to participants. Consequently, we do not perform diagnostic-group (CU/MCI/pAD) stratification in Apheleia-001; this limitation is discussed in the Limitations and Future Directions section.

### Digital clock and recall (DCR)

Linus Health's DCR (11–13) is an FDA-listed Class II software-as-a-medical device and a multimodal, ML–enabled evolution of the Mini-Cog (10). The three-minute assessment is completed on an iPad using an Apple Pencil and can be administered by medical or research assistants without specialized training.

The DCR is administered in three consecutive steps:

1. The assessment begins with an *Immediate Recall* task: three unrelated words are read aloud, and the participant repeats them to confirm encoding. Responses are spoken and recorded without a time limit. This step is not included in the overall DCR Score but helps assess attention/hearing and establishes a baseline for delayed recall.
2. Immediately after word encoding, the participant completes the *digital clock drawing test* [DCTclock™ (19, 33)] on an iPad in two sequential conditions. In the Command Clock condition, they draw a clock from memory, placing the numbers correctly and setting the hands to 11:10 without any template or visual cues. In the Copy Clock condition, they reproduce an on-screen clock set to the same time. Both drawings are completed with a stylus, while the software records the completion time and captures detailed drawing behavior data, along with the final images. Together, these tasks probe visuospatial and visuoconstructional ability, planning and executive function, processing speed, and motor control.
3. After the clock drawing tasks, the participant is asked to recall the original three words. Responses are spoken and recorded, while the application logs both response content and captures detailed acoustic and speech features. This *Delayed Recall* component assesses verbal episodic memory, which is often sensitive to early AD pathology, and completes the DCR administration.

### Data acquisition and feature extraction in the DCR

The DCR passively captures high-resolution drawing and speech data to derive a broad set of quantitative “process features.” During clock drawing, the system records time-stamped pen trajectories, enabling the extraction of velocity profiles, acceleration, stroke length, pen speed, total drawing duration, and time on-task vs. time off-tablet. It also derives temporal features, including pauses and latencies such as initiation time, inter-stroke intervals, and stage-specific latencies. From the final drawings, it computes spatial/organizational measures including clock face symmetry and circularity, numeral placement, clock hand placement, and proportions, and overall vertical and horizontal positioning on the screen (33, 34). In parallel, the DCR records spoken responses during recall to extract response latency, total duration, pauses, and acoustic features such as pitch, jitter, shimmer, and speaking rate (12, 13). Together, these modalities support the generation of roughly 2,000 features used by ML models for detection beyond traditional scoring.

### DCR scoring

DCR scoring is fully automated. The Mini-Cog-style (0–5) DCR score is derived from a weighted combination of clock-drawing and delayed-recall sub-scores and is expressed on a 0–5 integer tier with three clinical bands: Green (4–5) = Not Indicative of Cognitive Impairment, Yellow (2–3) = Borderline for Cognitive Impairment, and Red (0–1) = Indicative of Cognitive Impairment. Cohort-level summary statistics for the DCR total score (median and interquartile range [IQR]) have been added to Table 1, stratified by cognitive cohort, where available (CU/MCI/pAD in BH).

The immediate and delayed recall audio is transcribed via automatic speech recognition, and the recognized words are matched to the three target words for a 0–3 score (1 point per exact match). Immediate recall is recorded but not included in the 0–5 score, serving primarily as an encoding/attention/hearing check, and can be reviewed clinically.

The clock-drawing portion is scored automatically using a combined machine learning and rules-based engine. The system first “parses” the drawing by classifying each pen stroke into clock components (e.g., clock face, numerals, hour/minute hands, and extraneous marks/self-corrections) using both spatial layout and the temporal sequence of strokes. This stroke-level labeling, trained on large sets of annotated clocks and reinforced with hierarchical rules, supports robust scoring even for messy drawings and enables the extraction of cognitively informative timing and process metrics. Development, validation, and biomarker associations for the DCTclock are detailed in several prior studies (19–21, 33–38).

The DCR also generates an ML-derived score (0–100) to accurately identify cognitive impairment, computed from multimodal features captured during the DCR (13). The result is reported in three tiers: Green (60–100 score) indicates no significant impairment detected, Yellow (30–59 score) suggests borderline findings warranting monitoring or intervention, and Red (0–29 score) indicates a high likelihood of impairment consistent with MCI or dementia. The automated report presents the score and tier, along with playback and component-level breakdown to support rapid clinical interpretation and has been evaluated in multiple real-world implementation studies (23–27, 39–41).

### Analytical approach

We split participants into training (70%) and test (30%) sets, ensuring that the distribution of MMSE scores in each subset was similar to the full set (median = 27, SD = 2.81, kurtosis = −0.19). DCR features were automatically processed after each administration and included 307 drawing and speech DCR metrics (12, 13) plus age. Missing data (<2%) were imputed with the corresponding median values from the training set. We trained a weighted Poisson elastic net regression model (glmnet, via the glmnetUtils package (42)) on the 70% training partition. Ten-fold cross-validation was used solely to tune the elastic net hyperparameters [alpha (the L1 mixing ratio, between 0 and 1) and lambda (the overall regularization strength)], with the two parameters optimized simultaneously per fold. Once tuning was complete, a single model with the selected hyperparameters was fit on the full training partition; all reported performance metrics **(**RMSE, MAE, signed mean error, R^2^, and RCI) are computed on the independent 30% held-out test set using this single tuned model, not as averages across folds. For completeness, per-fold Poisson deviances (mean ± SD) is reported in the Supplementary Materials. All DCR features and age were included as potential predictors in the model. Given the sparse representation of low-MMSE scores in BH, we assigned additional weight to scores below 23, with the second-highest weight to scores above 27. The elastic net model, therefore, took the following form, where the Poisson regression was:

$$y_{i}∼\mathrm{Poisson}(\mu_{i})$$

$$\log(\mu_{i})\;=\;\beta_{o}\;+\;x_{i}^{T}\beta$$

where, for each participant i: yᵢ is the observed MMSE total score; *μ*ᵢ is the expected (mean) MMSE total score estimated by the model; *β*₀ is the intercept; *β* is the vector of regression coefficients (the feature weights to be estimated); xᵢ is the vector of predictor variables for participant i (the 307 DCR features plus age); and xᵢᵀ*β* is the linear predictor, i.e., the inner product of the predictors and their coefficients.

with the weight applied as

$$\ell (\beta_{o},\;\beta )\;=\;\Sigma_{i=1}^{n}\;w_{i}\;[\;y_{i}\;\log(\mu_{i})\;-\;\mu_{i}-\;\log(y_{i}!)]$$

In this expression, ℓ(*β*₀, *β*) is the weighted Poisson log-likelihood that the model maximizes; equivalently, −ℓ is the loss function to be minimized. *n* is the number of training participants, and the summation runs over all participants i = 1 … n; wᵢ is the observation weight assigned to participant i (defined below). The final term log(yᵢ!) is the natural logarithm of the factorial of yᵢ: the exclamation mark “!” denotes the factorial operation, so that yᵢ! = yᵢ × (yᵢ−1) × … × 2 × 1. This log-factorial term is the normalizing constant of the Poisson probability mass function; it does not depend on the model parameters and therefore does not affect the location of the optimum.

where weights wᵢ were chosen as the inverse of the rates of scores for each score range:

$$w_{i}=\left\{ \begin{array}{c} 2.0\;\;\mathrm{if}\;\mathrm{MMSE}\;\;<\;22\\ 1.0\;\;\mathrm{if}\;\mathrm{MMSE}\;\;<\;28\\ \;0.5\;\;\mathrm{otherwise} \end{array}\right.$$

The final form of the penalized log-likelihood minimized by glmnet was therefore:

$$\arg\;\min\;\beta_{o},\beta \;\{\;-\;\ell (\beta_{o},\;\beta )\;+\;\lambda \;[(1-\alpha )/2\;\|\beta {{\|}_{2}}^{2}\;+\;\alpha \;\|\beta {\|}_{1}]\;\}$$

Here, “arg min” over (*β*₀, *β*) denotes the intercept and coefficient values that minimize the penalized negative log-likelihood; *λ* ≥ 0 is the overall regularization strength (larger values shrink the coefficients more strongly); *α* ∈ [0, 1] is the elastic-net mixing parameter that balances the ridge (L2) and lasso (L1) penalties; ‖*β*‖₂² is the squared L2 norm of the coefficient vector (the sum of the squared coefficients); and ‖*β*‖_1_ is the L1 norm (the sum of the absolute values of the coefficients).

The resulting best model was validated on the test set using the root-mean-square error (RMSE) and mean absolute error (MAE) for predicted vs. observed MMSE total scores, both overall and stratified by demographics.

#### Reliable change index

We computed the Reliable Change Index (RCI) following the classical Jacobson–Truax formulation:

$$\mathrm{RCI}\;=\;(X_{2}\;-\;X_{1})\;/\;SE_{o}$$

where the standard error of the difference is given by

$$SE_{o}\;=\;S_{1}\;\surd 2\cdots \surd (1\;-\;r_{\mathrm{xx}}),$$

with X₁ and X₂ the two scores being compared (the observed and the model-predicted MMSE total score, respectively), S_1_ the standard deviation of the test score distribution, and r_xx_ the test–retest reliability coefficient. We used the MMSE test–retest reliability values reported by Tombaugh (2005) (6) for short inter-test intervals (<90 days). We did not apply a practice-effect correction because our “retest” is a single model-predicted score rather than a repeated human administration; hence, practice-induced shifts do not accumulate. For interpretability, we compare the RCI computed between our predicted and observed scores with Tombaugh's short-interval reference RCIs of 4.16 (T1–T2) and 4.01 (T3–T4), and, for reference, the broader long-interval range of 2.94–4.42.

For demographic subgroup analyses, we used percentile-bootstrap 95% confidence intervals (2,000 resamples) for each error metric and permutation tests (10,000 permutations) for between-group comparisons. These procedures do not require equal group sizes and are appropriate given the observed inequalities in ethnicity. We additionally report analogous age-band analyses (<60, 60–69, 70–79, 80–89, ≥90), flagging any band with *N* < 20 as exploratory.

## Results

The final optimized elastic net parameters were 0.21 for alpha (the L1 mixing ratio, between 0 and 1) and 2.98 for lambda (the overall regularization strength). The final 15 features selected by the model were related to delayed recall accuracy, total words produced during recall, speech production rates during immediate and delayed recall, clock-drawing features related to latency and component placement, and age. Poisson deviances for each cross-validation fold are reported in the Supplementary Materials.

In the BH data set, the DCR model predicted MMSE with an RMSE of 2.43 on the held-out test set (single-tuned model; not a CV average) (Figure 1A). Corresponding values of RMSE, MAE, signed mean error (predicted−observed), and R-squared for the BH test set, overall and stratified by Sex, Race, Ethnicity, and Age band, are reported in Table 2 with 95% bootstrap CIs. Based on (6), the short-interval RCI for MMSE is 4.16 (T1–T2) and 4.01 (T3–T4) for retests at <90 days, and the long-interval values range from 2.94 to 4.42. The observed-vs.-predicted RCI in the BH test set (4.23) was slightly above the short-interval reference values but within the broader long-interval range reported by Tombaugh (6), consistent with the predicted MMSE score being of comparable precision to a repeat short-interval human administration rather than strictly non-inferior. Only 6.4% of the predicted values were above this RCI threshold.

**Table 2:** Prediction evaluation metrics on the held-out BioHermes test set.

Variable	Level	n	RMSE (95% CI)	MAE (95% CI)	ME (95% CI)	R2 (95% CI)	Permutation (median difference; *p*-value)
All		282	2.43 [2.23, 2.64]	1.87 [1.69, 2.05]	−0.02 [−0.3, 0.26]	0.34 [0.24, 0.43]	NA
Sex	Female	176	2.39 [2.15, 2.61]	1.87 [1.66, 2.09]	0.19 [−0.18, 0.53]	0.38 [0.26, 0.49]	1.0; *p* > 0.8
	Male	106	2.51 [2.15, 2.85]	1.87 [1.58, 2.19]	−0.38 [−0.84, 0.08]	0.27 [0.13, 0.43]	
Age binned	<60	0	nan [nan, nan]	nan [nan, nan]	nan [nan, nan]	nan [nan, nan]	NA
	60–69	103	2.11 [1.77, 2.45]	1.52 [1.25, 1.83]	0.07 [−0.33, 0.5]	0.33 [0.15, 0.51]	
	70–79	135	2.43 [2.12, 2.72]	1.89 [1.62, 2.16]	0.04 [−0.37, 0.47]	0.32 [0.19, 0.45]	
	80–89	44	3.07 [2.58, 3.55]	2.61 [2.14, 3.11]	−0.43 [−1.3, 0.43]	0.19 [0.02, 0.43]	
	>=90	0	nan [nan, nan]	nan [nan, nan]	nan [nan, nan]	nan [nan, nan]	
Ethnicity	Hispanic	27	2.19 [1.74, 2.63]	1.85 [1.44, 2.33]	0.0 [−0.85, 0.89]	0.57 [0.39, 0.74]	−0.5; *p* > 0.99
	Non-Hispanic	246	2.45 [2.23, 2.67]	1.86 [1.67, 2.06]	0.01 [−0.29, 0.3]	0.3 [0.19, 0.4]	
	Unknown	9	2.67 [1.63, 3.61]	2.22 [1.33, 3.33]	−1.11 [−2.67, 0.56]	nan [nan, nan]	NA
Race	Non-White	38	2.25 [1.73, 2.77]	1.71 [1.26, 2.21]	−0.13 [−0.84, 0.61]	0.42 [0.17, 0.64]	1.0; *p* > 0.7
	White	244	2.46 [2.24, 2.67]	1.89 [1.7, 2.09]	−0.01 [−0.33, 0.3]	0.32 [0.22, 0.42]	

The top row reports overall performance; the remaining rows stratify the test set by sex, age band, ethnicity, and race. For each subgroup, we report sample size (n), root-mean-squared error (RMSE), mean absolute error (MAE), signed mean error (ME; predicted−observed), and R-squared, each accompanied by percentile-bootstrap 95% confidence intervals (2,000 resamples). Cells marked NA correspond to age bands with no participants in the test set. Performance was comparable across demographic subgroups and signed-ME confidence intervals spanned zero in every subgroup, indicating no systematic over- or under-prediction. The rightmost column reports between-group permutation tests on the median MAE for sex, ethnicity, and race (10,000 permutations); each entry reports the observed median MAE difference between groups, along with its two-sided *p*-value. Age bands were not compared this way; age-related model behavior is instead characterized in the Results through (i) a model-level RMSE comparison between models trained with vs. without age as a feature, and (ii) a linear regression of prediction residuals on age.

Models trained without age as an available feature achieved similar performance (RMSE = 2.28, MAE = 1.81), whereas an age-only model performed worse (RMSE = 2.78, MAE = 2.37). These analyses indicate that age alone does not account for the predicted score, supporting the model's capture of cognitively relevant process-based signals beyond demographic features. That said, including age in the model retains clinical benefits for physician usability.

We then performed external validation using the Apheleia data set, applying the pre-trained MMSE prediction model without any additional retraining. The previously trained DCR model predicted MMSE scores with an RMSE of 2.62 (Table 3). The corresponding RCI for Apheleia was lower than for Bio-Hermes at 3.4, on par with the slight increase in RMSE (indicating a more stringent reliable-change threshold given the cohort's restricted MMSE range), consistent with the modest increase in RMSE. This index is likewise interpreted against Tombaugh's short-interval reference values. Figure 1B is consistent with the absence of observed MMSE scores of 29–30 in this cohort, a structural feature attributable to Apheleia's cognitively-concerned inclusion criteria. Figure 1B shows that the relationship between observed and predicted scores was similar to the one in the BH dataset (Figure 1A).

**Table 3:** Prediction evaluation metrics for the independent Apheleia test set, with overall performance indicated on the top row (RMSE, root mean squared error; MAE, mean absolute error; ME, mean signed error; R2, R-squared).

Variable	Level	n	RMSE (95% CI)	MAE (95% CI)	ME (95% CI)	R2 (95% CI)	Permutation (median difference; *p*-value)
All		238	2.62 [2.38, 2.86]	2.08 [1.89, 2.28]	−0.86 [−1.16, −0.55]	0.18 [0.09, 0.28]	NA
Sex	Female	155	2.25 [2.03, 2.47]	1.81 [1.6, 2.03]	−0.45 [−0.78, −0.1]	0.26 [0.14, 0.39]	0.0; *p* > 0.99
	Male	83	3.19 [2.7, 3.7]	2.58 [2.18, 3.01]	−1.64 [−2.25, −1.05]	0.11 [0.01, 0.26]	
Age binned	<60	46	2.08 [1.68, 2.45]	1.72 [1.39, 2.07]	0.24 [−0.37, 0.83]	0.31 [0.09, 0.54]	NA
	60–69	83	2.25 [1.93, 2.56]	1.77 [1.47, 2.08]	−0.28 [−0.75, 0.23]	0.22 [0.05, 0.42]	
	70–79	53	2.7 [2.24, 3.16]	2.21 [1.77, 2.64]	−1.26 [−1.91, −0.62]	0.16 [0.02, 0.34]	
	80–89	56	3.34 [2.74, 3.96]	2.71 [2.25, 3.25]	−2.25 [−2.91, −1.64]	0.12 [0.01, 0.29]	
	>=90	0	nan [nan, nan]	nan [nan, nan]	nan [nan, nan]	nan [nan, nan]	
Ethnicity	Hispanic or Latino	46	2.32 [1.89, 2.71]	1.85 [1.46, 2.26]	−0.33 [−0.96, 0.33]	0.23 [0.03, 0.48]	−1.0; *p* > 0.10
	Not Hispanic or Latino	184	2.72 [2.42, 3.02]	2.17 [1.93, 2.42]	−1.03 [−1.4, −0.68]	0.17 [0.08, 0.27]	
	Not Reported	4	1.8 [0.0, 2.6]	1.25 [0.0, 2.5]	0.25 [−1.5, 2.25]	nan [nan, nan]	NA
	Unknown	4	1.66 [0.5, 2.65]	1.25 [0.25, 2.5]	−0.25 [−2.0, 1.0]	nan [nan, nan]	
Race	Non-White	56	2.6 [2.18, 2.99]	2.12 [1.75, 2.52]	−0.62 [−1.27, 0.0]	0.2 [0.05, 0.38]	0.0; *p* > 0.99
	White	182	2.62 [2.33, 2.92]	2.07 [1.84, 2.3]	−0.93 [−1.28, −0.57]	0.17 [0.08, 0.28]	

Each metric is paired with 95% bootstrap CIs based on 2,000 iterations. Results stratified by demographic are more variable than they were for the BioHermes cohort, with signed mean error confidence intervals suggesting underestimation for sex in particular. A permutation comparison on median absolute errors is presented in the last column for sex, ethnicity, and race (10,000 iterations). No comparisons were significant, but switching to a mean difference in the permutations did result in significant differences for sex.

Performance stratified by demographic characteristics showed similar trends to those from the BioHermes test set. RMSE, MAE, signed ME, and R^2^ with 95% bootstrap CIs are reported in Table 3. Between-group absolute error differences were assessed by median permutation tests (10,000 permutations) for all except age features (addressed via models on prediction errors further below). Full numerical values are reported in Table 3. No comparisons were significant, but we note that sex differed significantly when performing permutations on the mean (*p* < 0.001); this was not the case elsewhere*.* These results should be interpreted with caution, given the small subgroup sizes.

In addition to the pairwise demographic analyses reported above, we also examined whether prediction error is modulated by combinations of demographic variables. For each of the BH held-out test set and the Apheleia-001 cohort, we fit linear regressions of prediction residuals (predicted—observed MMSE). Raw coefficients, standard errors, CIs, and *p*-values are reported in Supplementary Table S2. A main negative effect of age was found for Apheleia (*T* = −5.41, *p* < 0.0001), as well as the previously observed significant difference in sex (*T* = −2.14, *p* < 0.05). However, adjusted R-squared values for these demographic models were low for both BioHermes (0.14) and Apheleia (0.005) cohorts.

**Figure 2:**
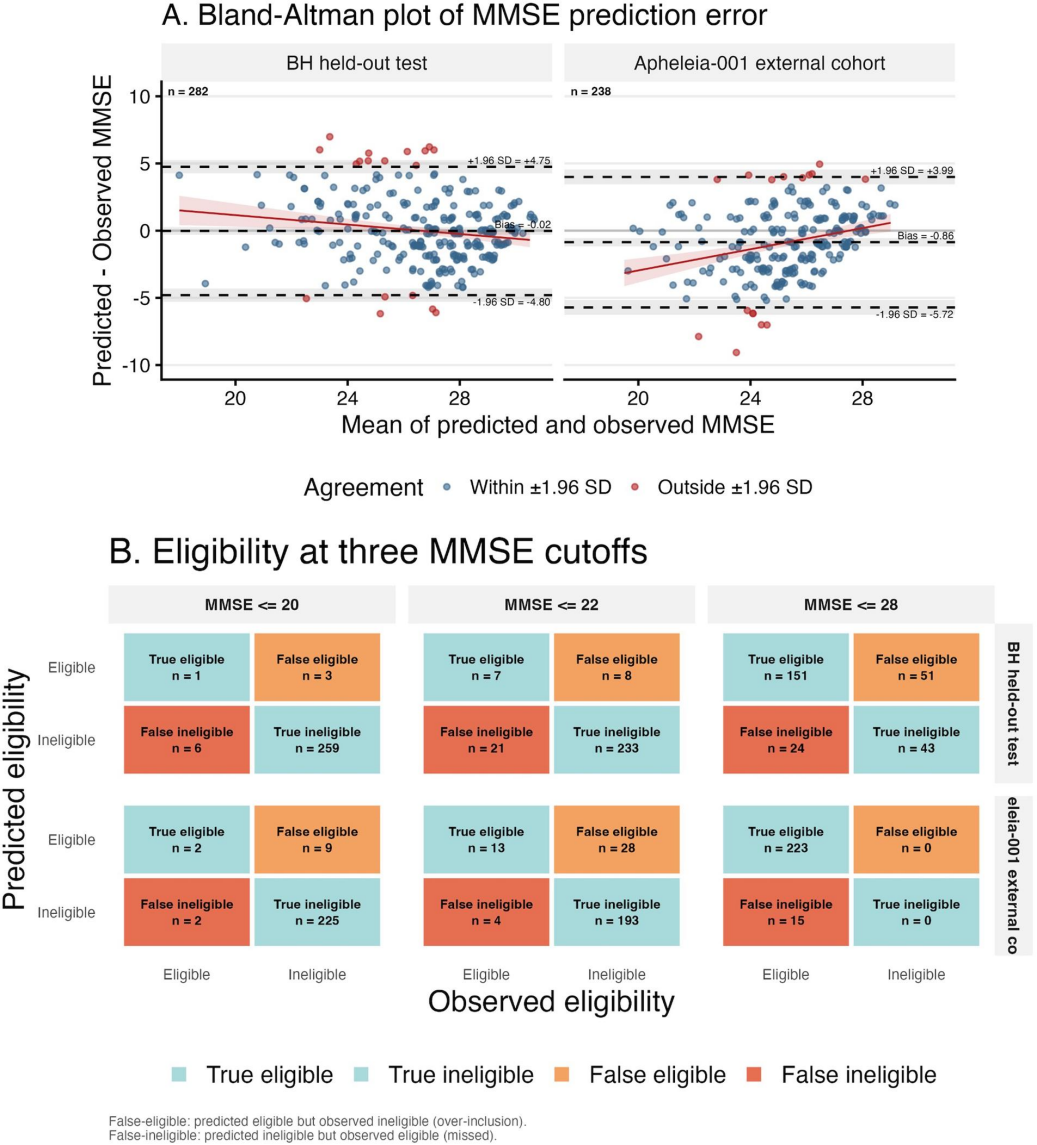
**(A)** Bland–Altman plots of MMSE prediction error (predicted−observed MMSE) plotted against the mean of predicted and observed MMSE, shown for the BH held-out test set (left) and the Apheleia-001 external cohort (right). Dashed lines mark the mean bias and the ±1.96 SD limits of agreement, with grey bands giving their 95% confidence intervals. The red line is an ordinary least-squares fit of the difference on the mean (a proportional-bias check), with its 95% CI shaded. Points within the ±1.96 SD limits are colored teal, and points outside red; horizontal jitter is applied to reduce overplotting at integer MMSE values. **(B)** Confusion matrices of predicted vs. observed eligibility at the three DMT-relevant MMSE cutoffs (≥20, ≥22, ≥28) for both cohorts. At each cutoff, a participant is classified as eligible if their MMSE is at or above the threshold; otherwise, they are classified as ineligible. Each cell reports the count of true-eligible, true-ineligible, false-eligible (predicted eligible but observed ineligible), or false-ineligible (predicted ineligible but observed eligible) classifications; correct classifications are colored teal/orange and misclassifications red.

Finally, because the clinical cost of over- vs. under-estimating MMSE is asymmetric near the DMT eligibility cutoffs, we report confusion matrices at MMSE ≥20, ≥22, and ≥28 for both cohorts (Figure 2B), together with a Bland–Altman plot of prediction error across the MMSE range (Figure 2A).

## Discussion

The principal finding of this study is that a machine learning model, leveraging multimodal process-based features from the Digital Clock and Recall (DCR), can predict MMSE scores with high accuracy (RMSE = 2.43). Additionally, predictive performance was consistent across key demographic groups, suggesting it was not materially influenced by demographic factors. This predictive capability holds important implications for the clinical implementation of novel anti-amyloid therapies. By accurately cross-walking digital motor-linguistic signals into a widely used cognitive staging measure, this approach offers a scalable solution to diagnostic bottlenecks that often delay patient access to disease-modifying therapies (DMTs).

### Benchmarking accuracy against the reference

To properly interpret the model's performance, it is necessary to contextualize the Root Mean Squared Error (RMSE) of 2.43. While the MMSE remains a key regulatory reference measure for determining eligibility for DMTs, it is psychometrically imperfect. Historical data on the test-retest reliability of the MMSE indicate that scores for the same individual can fluctuate by approximately 4 points at short inter-test intervals due to factors unrelated to clinical progression, such as administrator variability, practice effects, and time of day (6, 30). Consequently, our model's prediction error falls within the inherent margin of error of the MMSE test itself. This suggests that the digital prediction is of comparable precision to a repeat human administration of the MMSE and may function as an automated complementary rater while preserving the single-human-rater variance structure; the RCI analysis presented in Results contextualizes this claim against Tombaugh's short- and long-interval reference values (6).

### Addressing the implementation crisis for disease-modifying therapies

The FDA approvals of lecanemab and donanemab have created an urgent need for efficient patient identification. Current Appropriate Use Recommendations and payer coverage policies strictly limit these therapies to patients with MCI or early dementia, typically operationalized as an MMSE score of 22–30. This requirement has transformed the MMSE from a general screening tool into one of the primary gatekeepers for access (43, 44).

However, the DMT diagnostic pathway creates two related but distinct bottlenecks. First, health-system modeling by Mattke and colleagues projects that wait times for specialist evaluation and confirmatory biomarker testing (e.g., amyloid PET or cerebrospinal fluid analysis) could extend to approximately 50 months due to the shortage of behavioral neurologists and the surge in demand (45, 46). Second, separately, primary-care visits average less than 15 min (5, 47), which leaves little room for a 10–15-minute manual MMSE administration before any specialist referral can even be made. The DCR is aimed primarily at the second bottleneck: reducing the per-visit time burden of cognitive screening in primary care so that patients can be triaged into the specialist pathway more efficiently. Over that interval, an individual with Alzheimer's disease—whose MMSE typically declines by roughly two to four points per year (48)—could lose approximately 8–16 points, progressing from mild to moderate dementia (MMSE <22) and effectively “aging out” of eligibility before treatment can begin.

The DCR offers a “digital triage” mechanism to mitigate this crisis. Because the test can be self-administered in under 3 min by support staff in primary care—requiring no specialized training—it allows for high-throughput screening. By generating a predicted MMSE score immediately, the system can stratify patients who are likely in the “Goldilocks window” for treatment, e.g., recommended MMSE 22–30 for lecanemab (3) and MMSE 20–30 for donanemab (49), thereby prioritizing referrals for confirmatory biomarker testing (e.g., amyloid PET or CSF). This targeted approach optimizes specialist capacity, ensuring that scarce neurological resources are focused on patients most likely to qualify for intervention.

### Asymmetric clinical cost of prediction errors

Because DMT eligibility is gated at specific MMSE thresholds, an over-estimation of MMSE by the model carries a different clinical consequence than an equivalent under-estimation: over-estimation near the lower bound (e.g., MMSE 20/22) risks advancing a patient who is, in fact, out of the treatment window, whereas under-estimation near the upper bound (e.g., MMSE 28) risks excluding a patient who could still benefit. In our analyses (Table 2; Figure 2), we report signed mean error and confusion matrices at the ≥20, ≥22, and ≥28 cutoffs to make these asymmetries explicit. These metrics should be considered alongside RMSE/MAE when adopting the model for clinical triage; they also motivate our recommended deployment pattern, in which a predicted MMSE near a threshold triggers confirmatory standard administration rather than a direct eligibility decision.

### Mitigating systemic bias and promoting health equity

A further contribution of this study is to provide initial evidence of the demographic fairness of the DCR-based prediction model. The traditional MMSE is known to exhibit bias against individuals from underrepresented racial groups and those with lower educational attainment, often yielding false positives for impairment due to cultural or educational items rather than neuropathology (7–9, 50). Such biases in a gatekeeping instrument pose a risk of systemic exclusion, potentially denying life-altering therapies to non-White populations.

Our analysis of the BH sample provides preliminary evidence of comparable prediction performance across racial (White vs. Non-White) and ethnic (Hispanic vs. Non-Hispanic) subgroups on the held-out test partition. We note that because the BH held-out test set is drawn from the same enrollment as the training data, training-distribution effects on subgroup performance cannot be fully excluded from this analysis alone. For this reason, we also computed the same stratified performance in the independent Apheleia-001 cohort (see Results and Table 3), where a comparable pattern was observed, strengthening but not conclusively establishing the claim of demographic fairness. This stability likely stems from the DCR's reliance on *process-based* features—such as drawing/thinking speed, latency, and motor kinematics—which are more biologically grounded and less culturally mediated than the *content-based* knowledge questions of pen-and-paper tests (11, 14, 15). By filtering out sociodemographic noise, the digital model may provide a more equitable estimate of global cognition, supporting the fair allocation of emerging therapies.

### Demographic effects

Although demographic comparisons of prediction error were small in both cohorts, fairness arguments are strengthened by also considering whether effects of demographic variables persist when examined concurrently. Accordingly, linear model analyses on the prediction residuals are reported in the Results and Supplementary Table S2. Besides a near-threshold significant difference for Sex in the Apheleia cohort, we only found that prediction errors show a significant age-related trend in the Apheleia cohort, with signed residuals becoming progressively more negative at older ages (indicating greater under-prediction in older participants); this pattern warrants caution near the lower DMT eligibility thresholds and motivates further investigation in larger, age-diverse samples. These findings extend, rather than replace, the permutation demographic-fairness analyses: they are consistent with the interpretation that the DCR-based crosswalk does not exhibit appreciable differential prediction error across demographic combinations within the observed cohorts. Because subgroup sample sizes for the three-way contrast are modest, we consider these interaction results as exploratory and as a foundation for confirmatory replication in larger, more demographically diverse samples.

### Advantages over traditional testing

Beyond accurately estimating the MMSE score, the DCR-based approach offers distinct clinical advantages.

- **Sensitivity to Subtle Change:** A prior analysis of the BH dataset found that the DCR is more sensitive than the MMSE for detecting cognitive impairment, identifying subtle impairment even among individuals who score at or near the ceiling on the MMSE (11).
- **Operational Efficiency:** The integration of automated scoring into the electronic health record (EHR) removes the administrative burden of manual scoring and documentation, addressing a primary barrier to cognitive assessment reported by primary care physicians (47). It also mitigates the effects of subjective administration and scoring.
- **Longitudinal Monitoring:** The continuous, granular nature of digital metrics captured by the DCR allows for more precise monitoring of disease trajectory, treatment response, or adverse events than coarse integer-scale tests (20, 43).

### Limitations

This study has some limitations. The analysis is cross-sectional; longitudinal validation is required to confirm that changes in predicted MMSE scores track accurately with disease progression and treatment effects over time. Additionally, while the Apheleia cohort provided external validation, further testing in broader community-based primary care populations is necessary to ensure generalizability outside of research-interested volunteers. We further note that the Apheleia-001 cohort is a cognitively-concerned/memory-complaint sample by design and therefore (i) lacks a true cognitively-unimpaired population (no observed MMSE 29–30) and (ii) does not carry diagnostic (CU/MCI/pAD) labels, which precludes diagnostic-group stratification in the external cohort. Both our Apheleia and BH-test subgroup analyses by sex, race, and ethnicity use bootstrap CIs, permutation tests, and main-effect-oriented linear models to accommodate modest subgroup sizes; the resulting CIs, while informative, remain wider than those from the larger BH cohort, and we flag small subgroup estimates as exploratory throughout. The same limitation precluded our ability to meaningfully explore interaction effects across demographic characteristics. Most importantly, both Bio-Hermes-001 and Apheleia-001 are cross-sectional by design, and each participant in the present analyses contributed a single paired DCR + MMSE assessment. The results reported here, therefore, establish concurrent agreement between predicted and observed MMSE rather than longitudinal sensitivity to within-person cognitive change. Accordingly, the present findings should be interpreted as evidence of cross-sectional crosswalk validity, not of longitudinal predictive validity over time, and any clinical use of the predicted score for tracking individual trajectories should be reserved until the longitudinal validity of this model has been established.

### Future directions

To address the cross-sectional limitation noted above, future work should evaluate the model's longitudinal predictive validity using suitable longitudinal datasets as they become available. Such evaluations could include several pre-specified analyses: (a) within-person change correlation between predicted and observed MMSE across visits (Pearson r and Spearman *ρ* on *Δ*-scores, with 95% bootstrap CIs); (b) longitudinal RCI agreement, applying the Jacobson–Truax RCI to predicted-vs-observed change scores using Tombaugh (6) reference values for the appropriate inter-test interval; and (c) time-to-threshold-crossing analyses for the DMT eligibility cutoffs (MMSE ≥20, ≥22, and ≥28), comparing the time at which a participant's predicted MMSE first crosses each threshold to the time at which the observed MMSE does so, using survival-style methods. Together, such analyses would determine whether the present cross-sectional crosswalk also tracks within-person cognitive change with sufficient fidelity to support longitudinal clinical decision-making.

## Conclusion

Machine learning applied to multimodal, process-based features of DCR performance can accurately and equitably predict MMSE scores, providing a scalable proxy for traditional testing. As healthcare systems adapt to the era of disease-modifying Alzheimer's treatments, this tool offers a critical mechanism to streamline eligibility assessment, reduce specialist bottlenecks, and ensure that access to therapy is determined by cognitive impairment and pathology rather than demography.

## Data Availability

The data underlying the findings of this study were collected as part of the Bio-Hermes-001 study (ClinicalTrials.gov ID: NCT04733989) and Apheleia-001 study (ClinicalTrials.gov ID: NCT05364307) and are governed by the Global Alzheimer's Platform Foundation (GAP) consortium agreement. The Bio-Hermes data, and potentially the Apheleia data, will be made available in the future via the Alzheimer's Disease Data Initiative (ADDI) AD Workbench (https://www.alzheimersdata.org) at the discretion of GAP. All requests for data access should be made directly to GAP at info@globalalzplatform.org. The code used to calculate the reported results is available from Linus Health, Inc. upon reasonable request and with the permission of Linus Health, Inc. (requests to: info@linus.health).
