# Supplementary material for: Streamlining eligibility assessment for Alzheimer's disease-modifying therapies: Prediction of MMSE scores using the digital clock and recall
Source: Front Digit Health. 2026 Jul 9;8:1799372. doi: 10.3389/fdgth.2026.1799372 (PMC13391514; doi:10.3389/fdgth.2026.1799372)
Supplement: Supplementary file 1 [file Table1.docx]

| **Fold** | **Mean Deviance** | **SD Deviance** |
| --- | --- | --- |
| 1 | 0.33 | 0.06 |
| 2 | 0.44 | 0.19 |
| 3 | 0.40 | 0.24 |
| 4 | 0.37 | 0.11 |
| 5 | 0.43 | 0.26 |
| 6 | 0.25 | 0.06 |
| 7 | 0.42 | 0.20 |
| 8 | 0.35 | 0.14 |
| 9 | 0.25 | 0.10 |
| 10 | 0.42 | 0.17 |

**Table S1.** Mean and SD deviances across lambdas and alphas for each fold of the fitted cross-validated Poisson elastic net model. Deviance was used instead of RMSE for model selection, and RMSE was then used as the evaluation metric on held-out and external test sets.

| **Cohort** | **Term** | **Coefficient** | **SE** | **T-value** | **p-value** | **Low 95% CI** | **High 95% CI** |
| --- | --- | --- | --- | --- | --- | --- | --- |
| BioHermes | Intercept | 1.67 | 1.61 | 1.03 | 0.30 | -1.51 | 4.86 |
|  | Sex (Male) | -0.54 | 0.30 | -1.81 | 0.07 | -1.14 | 0.04 |
|  | Race (White) | 0.29 | 0.43 | 0.67 | 0.50 | -0.56 | 1.14 |
|  | Ethnicity (Non-Hispanic) | 0.00 | 0.49 | 0.01 | 0.98 | -0.97 | 0.98 |
|  | Age | -0.02 | 0.02 | -1.09 | 0.27 | -0.06 | 0.01 |
| Apheleia | Intercept | 5.27 | 1.05 | 5.01 | < 0.01 | 3.20 | 7.34 |
|  | Sex (Male) | -0.69 | 0.32 | -2.14 | < 0.05 | -1.34 | -0.05 |
|  | Ethnicity (Non-Hispanic) | -0.46 | 0.38 | -1.21 | 0.22 | -1.21 | 0.29 |
|  | Age | -0.07 | 0.01 | -5.41 | < 0.001 | -0.10 | -0.05 |

**Table S2.** Results from linear models on model prediction errors for BioHermes-test and Apheleia data sets. Each model includes available demographic information, with age as a continuous variable (compared to a bin in previous analyses). BioHermes had no significant effects. For Apheleia, we found the same sex-wise significant difference in mean absolute errors, as well as a main effect of age (although this effect was negative). Adjusted R-squared values for these demographic models were low for both BioHermes (0.14) and Apheleia (0.005) cohorts.
